# Blue and fin whales in the Northern Mariana Islands: Their call characteristics and occurrence

**DOI:** 10.1371/journal.pone.0329398

**Published:** 2025-08-21

**Authors:** Camille Ollier, Megan Wood, Erin M. Oleson, Ana Širović

**Affiliations:** 1 Centre d’Etude Biologiques de Chizé, UMR 7372 CNRS – La Rochelle Université, Chizé, France; 2 Observatoire PELAGIS, UAR 3462 CNRS – La Rochelle Université, La Rochelle, France; 3 Saltwater, Inc, Anchorage, Alaska, United States of America; 4 Pacific Island Fisheries Science Center, NOAA Fisheries, Honolulu, Hawaii, United States of America; 5 Trondhjem Biological Station, Department of Biology, Norwegian University of Science and Technology, Trondheim, Norway; Kobe University, JAPAN

## Abstract

Many baleen whale vocalizations are species-, or even population-specific and can be used to monitor their occurrence. Although baleen whale occurrence has been well studied in parts of the Pacific Ocean, little is known about the seasonal distribution of blue and fin whales in the Western Pacific. Since 2010, a more concerted visual and acoustic survey effort has occurred around a very remote region of the western Pacific Ocean: the Northern Mariana Islands. Passive acoustic data were collected at two locations in the Northern Mariana Islands, Saipan and Tinian, from 2015–2017 and they were analyzed for call characteristics and occurrence of blue whale and fin whale calls. Low levels of Central North Pacific blue whale tonal calls were detected year-round with peaks in the winter (December) and summer (June). There was a clear seasonal pattern in fin whale calls (20 and 40 Hz calls), with the majority of detections occurring during winter and spring. Moreover, two unknown low-frequency sounds were detected, one tonal and one pulsed. The former was more common at Tinian and the latter at Saipan. By providing their acoustic features and occurrence patterns, we aim to facilitate future identification of their source. Additionally, the observed seasonal patterns in blue and fin whale call occurrences may offer insights into their movement patterns in this remote region.

## Introduction

Many baleen whales spend summers foraging at high latitudes where food is more abundant and then migrate several thousand kilometers during the fall to spend the winter in warm water, low latitude breeding grounds [1,2]. The timing of seasonal migration may depend on prey availability and environmental factors [3–4] and varies among species. Some species travel shorter distances, such as sei whales (*Balaenoptera borealis*) [5–7], or maintain year-round residency like some populations of Bryde’s whales (*B. edeni*) [5]. Blue whales (*B. musculus*) and many populations of fin whales (*B. physalus*), on the other hand, undergo these large seasonal migrations.

Baleen whale occurrence has been studied relatively well in the eastern North Pacific (ENP) Ocean [2,8–14] but less is known about their seasonal distribution in the western North Pacific Ocean. The region near the Northern Mariana Islands, next to the Mariana Trench, the deepest zone in the ocean [15], is known to be inhabited by blue whales, fin whales, sei whales, minke whales (*B. acutorostrata*), Bryde’s whales and humpback whales (*Megaptera novaeangliae*) [16]. Most visual and acoustic effort to understand baleen whale occurrence off the Mariana Islands has occurred since 2010 [16,17]. Small vessel nearshore visual surveys were typically conducted in the spring or summer annually, from 2010 to 2019. Additionally, long-term passive acoustic monitoring near Saipan started in 2010. Blue and fin whales have not been sighted during visual surveys [16,17], but some preliminary acoustic analyses indicated acoustic detections occurred mostly in the winter and spring, although they were not very frequent [16]. Humpback whale seasonal occurrence has been documented with sightings and acoustic records in the winter and early spring [17,18]. Bryde’s whales were visually detected in most months of the year, and acoustic records also included unidentified calls that may belong to Bryde’s whales [16].

Blue and fin whales are well suited for acoustic studies of their occurrence due to the low frequency and high intensity of their calls [19]. Blue whales emit two categories of sounds: song and non-song. The first are relatively long (up to 10 s) and low-frequency (<200 Hz) sounds. Males are known to produce these units at regular intervals (termed song) and they are thought to serve a reproductive function [20]. Blue whale songs comprise at least eleven regionally distinct dialects worldwide [21,22]. Blue whales also produce a shorter (1–4 s) downswept call type, the D call, which has been documented in irregular intervals [23] and mostly occurs during the spring and early summer in the ENP [13,24]. The call occurs when blue whales are engaged in foraging activities and most likely functions in a socializing context [20]. Blue whale D calls are thought to have the same characteristics across blue whale populations and therefore are not used for population structure studies [25,26]. Additional evidence indicates that D calls play a role across populations and may function as contact calls in different social contexts [27].

At least three blue whale song types exist in the North Pacific Ocean [21,22]. The first is the ENP song which has been well documented in the ENP blue whale population. It generally consists of two distinct units, A and B, repeated in sequences [20,21,23]. These blue whale song units have been detected during the summer and fall in their feeding areas from California to the Gulf of Alaska and in the winter in the breeding areas in the Eastern Tropical Pacific (ETP) [11,14,28–32]. The second blue whale song type in the North Pacific Ocean is the Central North Pacific (CNP) song which consists of a simple tonal unit downswept from 20 Hz to 18 Hz lasting more than 8 s [30,33,34]. Blue whales in the Gulf of Alaska produce these tonal calls mainly in August and September [11,30]. Presence of these calls was also detected in the summer in the western North Pacific [22]. The third likely blue whale song in the North Pacific was recently identified off Japan, which consists of a long (>10 s) call that downsweeps from about 19–16 Hz, often followed by a shorter tonal near 14 Hz [35]. This “Japan song” was recorded off Hokkaido in January and September [35].

Fin whales also produce two well documented types of calls, known as “20 Hz” [36] and “40 Hz” calls [36,37]. The “20 Hz*”* call has been reported worldwide. It is known to occur at regular intervals; time intervals between individual 20 Hz pulses and the frequency range of a higher frequency component are regionally distinct [38–41]. These calls, recorded in the North Pacific, do not feature a higher frequency component and occur year-round with a peak in autumn and winter in the ENP [14,30,42,43]. They may be associated with a mating function when produced in regular sequences [37,44,45]. When produced at irregular sequences, they are likely used for social purpose [46]. The “40 Hz” call is a short duration call [37] generally produced at irregular intervals [36] and may be less ubiquitous. These calls appear to be common during the summer in the ENP and their purpose may be related to foraging [37]. Report from Hill et *al.* [16] shows that fin whale occurrence is not common in the Mariana Archipelago, but calls are recorded through winter and spring.

The understanding of baleen whale call repertoires remains uncertain for species and populations occurring in more remote regions. In this paper, we present the results of blue and fin whale presence from the analyses of two years of passive acoustic data collected from 2015 to 2017, off Saipan and Tinian. We also describe two previously undescribed signals resembling baleen whale vocalizations that were detected year-round at both sites, but could not be attributed to a vocalizing species. By analyzing call characteristics and occurrence, we provide a better understanding of the species’ vocal repertoire and their presence in this area.

## Materials and methods

**Fig 1:**
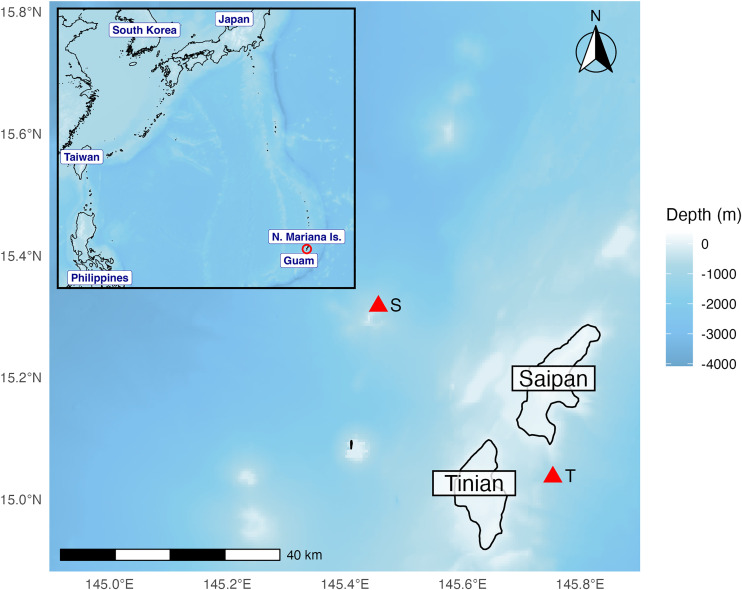
Map of hydrophones deployment locations. Hydrophone deployment locations (red triangles) on the west side of Saipan (marked with S) and on the east side of Tinian (marked with T) in western Pacific Ocean. Color bar indicates depths in meters. Map generated using ggplot2 package in R [48] and bathymetry data from the ETOPO2022 database hosted by NOAA [49].

Passive acoustic data were collected in the Mariana Archipelago, in the western Pacific Ocean. High frequency Acoustic Recording Packages, HARPs, [47] were deployed at two recording stations in the archipelago, one west of Saipan and the other east of Tinian (Fig 1).

HARPs sampled at 200 kHz on a 5-minute duty cycle over a 7 min interval from May 2015 to May 2017 in Saipan and May 2015 to November 2016 in Tinian (Table 1). Upon recovery, raw data were decompressed and converted into WAV audio files for easier analysis. The data were decimated by a factor of 100 to create a dataset with an effective bandwidth up to 1 kHz to enable faster analysis of baleen whale signals. Long-Term Spectral Averages (LTSAs) were computed from these data by calculating 5 s spectra with 1 Hz frequency resolution.

**Table 1:** Deployment information for the HARPs located in Northern Mariana Islands.

Site name	Recorder latitude (N) and longitude (E)	Recording start and end dates (mm/dd/yyyy)	Depth (m)
Tinian	15°02.32’ 145°45.30’	5/13/2015-5/23/2016 5/30/2016-11/05/2016	1,000
Saipan	15°19.05’ 145°27.43’	5/13/2015-5/02/2016 5/30/2016-5/17/2017	600

Analysis of call characteristics and presence was performed manually using Triton (version 1.93), a custom Matlab-based software. Long Term Spectral Averages (LTSAs) were reviewed in one-hour windows over 0–250 Hz frequency band providing an efficient way to scan the long-term recordings for baleen whale calls (S1 Fig). Analysis effort for this study was conducted for blue whale CNP song and Japan song types, fin whale 20 Hz and 40 Hz calls, and any regular low-frequency tonal or pulsed signal of unknown origin. When signals of interest were visible in the LTSA, the corresponding periods were zoomed in on to review via a spectrogram and audio playback which was used when necessary to identify the call type. Each identified call was then individually logged in Triton. The spectrogram window was set as a frequency display of 1**–**150 Hz with a 60 second plot length. The FFT (Fast Fourier Transform) was calculated using 2,000 data points with a 90% overlap to create spectrograms of 0.1s temporal resolution. For each call logged, minimum and maximum call frequencies, and call start and end times, were measured from spectrogram and time series plots. The duration of calls was also calculated. All the information extracted for each call was saved into an Excel spreadsheet. As the intention of the study was to describe call type characteristics produced by baleen whales in this area as well as the call occurrence over time, mean values and associated standard deviations were calculated for the measured minimum and maximum frequency and overall duration of each type of call.

Acoustic datasets often contain periods with high background sound level that can mask possible signals of interest for different lengths of time. Due to low and variable detectability during such noisy periods, each 5 min duty cycle that had any duration of background noise likely to mask low-frequency signals of interest in the LTSA was logged as off effort and no calls that occurred during those 5 min were logged. Recording effort for each day was thus calculated by summing up all daily 5 min periods with on effort logging.

### Inclusivity in global research

Additional information regarding the ethical, cultural, and scientific considerations specific to inclusivity in global research is included in the Supporting Information (S1 Checklist).

## Results

A total of five distinct low-frequency call types were identified in the recordings from Saipan and Tinian. Three of them were known baleen whale call types: CNP tonal from blue whales*,* and 20 Hz and 40 Hz from fin whales. The remaining two call types resembled baleen whale calls, but differed in frequency, so they were classified: Unidentified tonal and Unidentified pulsed sound.

### Blue whales

**Fig 2:**
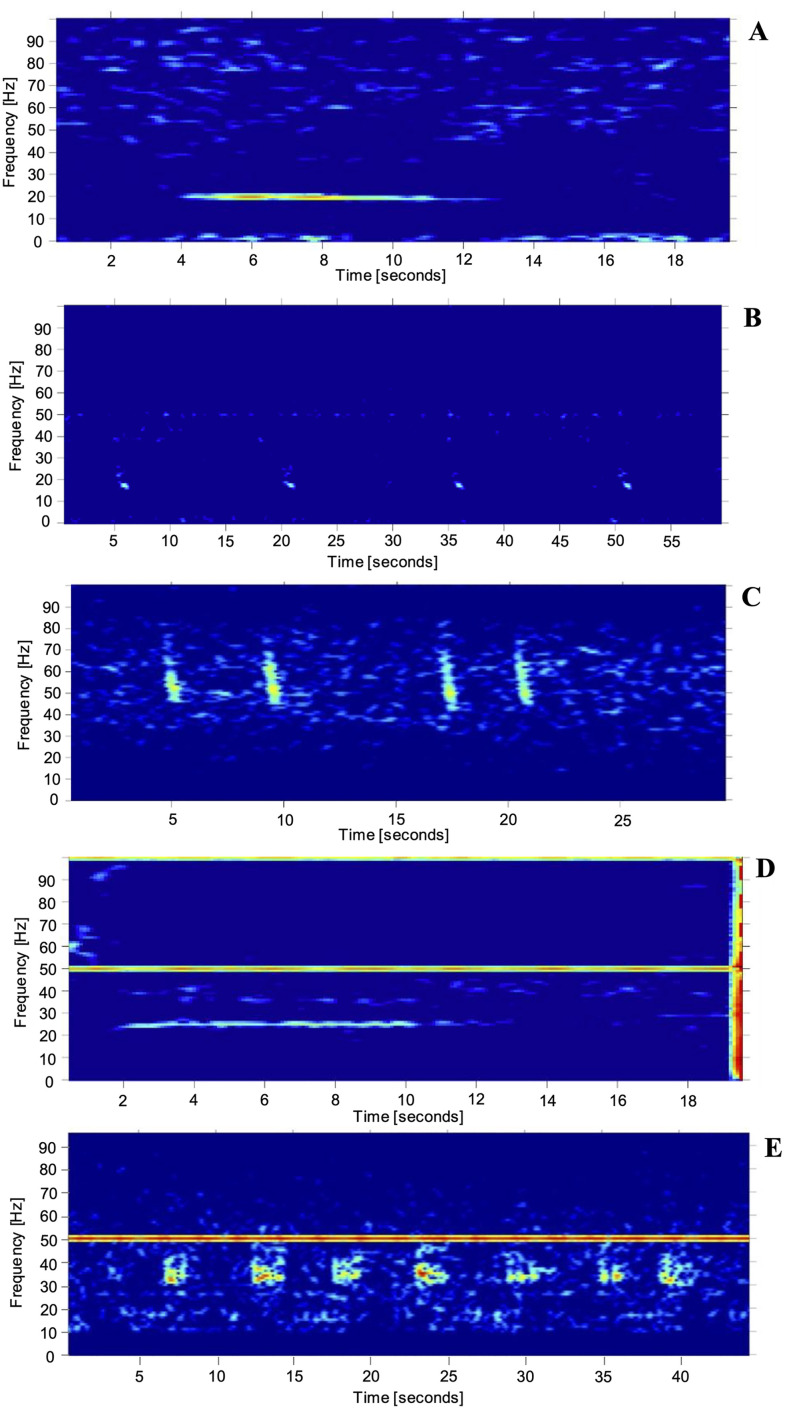
Spectrograms of baleen whale calls recorded in Saipan and Tinian. (A) Central North Pacific tonal blue whale call recorded at Tinian. (B) Fin whale 20 Hz call recorded in Tinian. (C) Fin whale 40 Hz call recorded in Saipan. (D) Unidentified tonal sound recorded in Tinian. (E) Unidentified pulsed sound recorded in Tinian. Spectrograms were created with 2000-point FFT with 90% overlap. The line at 50 Hz visible in some spectrograms represents instrument self noise (disk write).

Among blue whale songs, only the CNP tonal calls were identified in these recordings; no instances of Japanese song were observed. These calls consisted of a long tone (hence also the name CNP tonal call) starting at 21 Hz and slowly decreasing to 17 Hz. They lasted on average 6.6 seconds (Table 2, Fig 2A).

**Table 2:** Summary statistics for the acoustic characteristics of call types logged in Saipan and Tinian. Start, End frequency (Hz) and duration (s) ± standard deviation of each characteristic call type is shown, as well as the total number of calls that measurements are based on.

Species	Call type	Start Frequency (Hz)	End Frequency (Hz)	Duration (s)	Number of logged calls
Blue whale	Central North Pacific tonal	21 ± 1	17 ± 2	6.6 ± 7.8	274
Fin whale	20 Hz call	22 ± 3	14 ±2	1.2 ± 0.4	8009
	40 Hz call	74 ± 13	51 ± 8	0.8 ± 0.2	508
Unidentified whale	Tonal sound	29 ± 2	24 ± 2	5.5 ± 1.6	149
	Pulsed sound	62 ± 12	48 ± 9	0.7 ± 0.1	80

**Fig 3:**
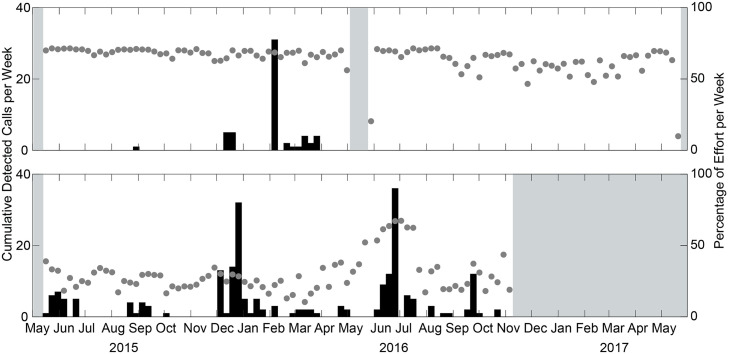
Weekly detections of CNP blue whale tonal calls at Saipan (top) and Tinian (bottom). Gray dots represent weekly recording effort including accounting for periods of high noise and grey shaded areas represent periods with no recording.

A total of 274 blue whale calls were detected; 56 of those calls were detected at Saipan and 218 at Tinian. The CNP tonal was detected only in the first year of data at Saipan with peak detection in February 2016 (Fig 3). The call was much more common at Tinian, with low-level of detections year-round and peaks in winter (December) and summer (June). The summer peak coincided with the period of higher effort.

### Fin whales

Two fin whale call types were detected in the two-year dataset in Tinian and Saipan: 20 Hz call and 40 Hz call. All calls were short duration but differed in frequency. The 20 Hz was a regularly repeated call, sweeping down from 22 to 14 Hz and lasting 1.2 seconds (Table 2; Fig 2B). It did not have a higher frequency component that is seen from fin whales in some regions. The 40 Hz call swept down from 74 to 51 Hz with a duration of 0.8 seconds. The 40 Hz call occurred in irregular sequences (Table 2; Fig 2C).

**Fig 4:**
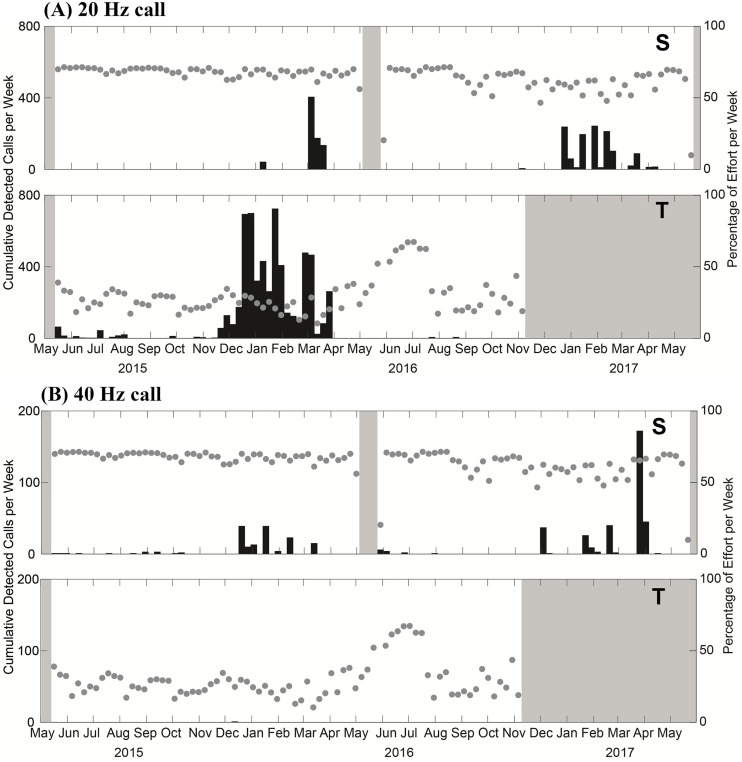
Weekly detections of (A) 20 Hz and (B) 40 Hz calls at Saipan (marked S) and Tinian (marked T). Gray dots represent weekly recording effort including accounting for periods of high noise and grey shaded areas represent periods with no recording.

Fin whale call occurrence showed a seasonal presence with peak call detections in winter. The 20 Hz call type was the most prevalent fin whale call detected. It was detected three times more frequently at Tinian (n = 6033) than in Saipan (n = 1976). Nearly all fin whale 20 Hz calls were detected from December to March at these locations (Fig 4A). Unlike the 20 Hz call, more 40 Hz calls were detected at Saipan (1 at Tinian and 507 at Saipan). The 40 Hz calls occurred also primarily in the winter and spring, between December and April, (Fig 4B).

### Unidentified calls

Two unknown call types: unidentified tonal and unidentified pulsed sound, were detected in the acoustic datasets and were assumed to be most likely produced by a cetacean species due to their low frequency*.* The tonal sound had a distinctively higher frequency than the CNP blue whale tonal hence its identification as a unique call type. The sound started at 29 Hz and ended at 24 Hz with a mean duration of 5.5 seconds (Table 2; Fig 2D).

The pulsed sound was different from other call types reported in the study. It consisted of a series of low-frequency repeated pulses (Fig 2E). Typically, the pulses ranged from 62 to 48 Hz. Each pulse sequence lasted on average 5.5 seconds (Table 2).

**Fig 5:**
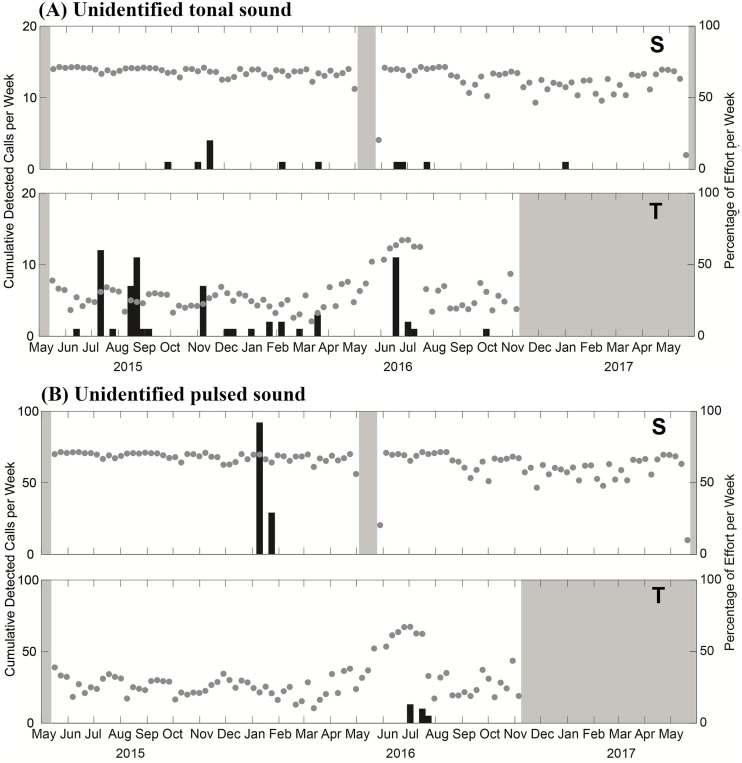
Weekly detections of (A) unidentified tonal sound and (B) unidentified pulsed sounds at Saipan (marked S) and Tinian (marked T). Gray dots represent weekly recording effort, including periods of high noise, and grey shaded areas represent periods with no recording.

The unidentified tonal and pulsed sounds were identified 80 and 149 times, respectively, across the two locations. The tonal sound was detected rarely in Saipan and it occurred irregularly throughout the duration of the acoustic recordings, with higher occurrences in the summer in Tinian (Fig 5A). The pulsed sound was detected in January 2016 in Saipan and in July 2016 in Tinian (Fig 5B).

## Discussion

We identified three known baleen whale call types and two additional sounds that are likely produced by baleen whales in recordings off Saipan and Tinian from 2015 to 2017. Fin whale calls were the most common and were mostly detected in the winter. The blue whale CNP tonal had a peak in winter months at both sites, with a second peak in summer occurring only at the Tinian site. Interestingly, occurrence of blue and fin whales was higher at Tinian than Saipan. Given the location of the recorder in Tinian to the east of the island, that may indicate that those animals do not often range into the Philippine Sea or, alternatively, could reflect differences in propagation due to oceanographic features.

### Blue whale

The blue whale call repertoire has been extended since McDonald and colleagues [21] identified nine populations-specific songs, with a recent study recognizing up to eleven. [22]. The blue whale call recorded in this study was a simple tonal call unit similar to those previously reported in the central and western Pacific, CNP blue whale tonal. The tonal call described by Širović et *al.* [33] tends to last more than 8 seconds while the tonal we measured here was just shorter than 7 seconds, albeit with a large standard error. Generally, detected calls were faint, indicating distant whales. Thus, the variation in call duration could be due to the long propagation from the sound-producing whale to the recording hydrophone, resulting in constructive and destructive interference of multiple arrivals of this call. Recordings of higher intensity signals would be important for providing a more definitive description of the acoustic features of this blue whale song unit.

The difference in acoustic occurrence of blue whales off Saipan and Tinian, with peak call detections in the winter at both sites and just off Tinian in the summer, are likely the result of the geographic location of the recorders relative to the islands. The Saipan recorder was deployed to the west of the island and monitors the basin of the Philippine Sea. Its detection range for blue and fin whale calls was likely in the range of 10 s to over 100 km (Širović [Unpublished]). The Tinian recorder, on the other hand, was to the east of the island, thus recording sounds from the western Pacific Ocean basin, but also likely detecting blue and fin whale calls out to distance of 100 km (Širović [Unpublished]). Previous acoustic studies document the presence of blue whale D calls in May and June 2014 [16] and visual observations identified blue whales during summer 1992 around Wake Island, located approximately 2,200 km west of the Northern Mariana Islands [31]. These results likely point to the fact that parts of this CNP blue whale population are found across the Western Tropical Pacific (WTP) in the summer but probably do not enter the Philippine Sea.

The bimodal presence of CNP tonal in the WTP, with peaks in winter and summer, provides a counterpoint to the seasonal presence of this blue whale song in the Gulf of Alaska where it peaks in the summer months of July and August [11,30]. Where this population migrates to breed in the winter is still unknown, as calls similar to the CNP tonal were rare in the ETP, in the WNP, and north of Hawaii [34,50]. The results of this study could indicate that this region may be part of the migration corridor between higher-latitude feeding grounds off Alaska and lower-latitude breeding grounds. While CNP song was observed to peak in winter in this study, calls were still relatively rare and faint. Overall, these data extend the geographic range of this call type into the WTP in addition to the Gulf of Alaska, with occasional visitation east to the ETP, where they might overlap with ENP blue whale population [51]. To better understand their full range, more acoustic effort is needed across this region especially to identify where this population occurs during the breeding season.

We did not detect the Japan-type song during our two years of study off Saipan and Tinian, leading us to hypothesize that blue whales producing this song do not regularly visit this region to the south. To date, the Japanese song has not been reported anywhere else.

### Fin whale

Two types of fin whale calls were detected in Saipan and Tinian: 20 Hz and 40 Hz calls. The 20 Hz call is typically the most common call produced by fin whales recorded in all major oceans [45]. The downswept 20 Hz calls we found were generally repetitive, representing stereotypical 20 Hz fin whale song [52]. The 40 Hz call characteristics we measured were very similar to those reported previously [37]. While there can be concerns about distinguishing between blue whale D calls and fin whale 40 Hz calls due to their similar frequency characteristics [20,37], in this study we used the short duration of 40 Hz calls (~1 s) as a classifying feature during logging.

A clear seasonal trend in daily call detections was identified in both locations where most of the calls occurred in winter and spring seasons. These results are consistent with past reports that documented 20 Hz calls to occur seasonally near Saipan and Tinian [16,17]. In the fall, when fin whale acoustic occurrence was not documented in Saipan and Tinian, they were heard close to the Emperor seamount chain in 1991 [53]. Fin whale 20 Hz calls were also observed in the northwest Pacific between 2000 and 2014 [54]. Given the typical winter-spring occurrence of the fin whale call in these waters, we can hypothesize that this fin whale population might migrate between the WTP waters where they spend winter and spring and another location during summer and fall.

### Unknown calls

Finally, we recorded two low-frequency sounds with unknown origin. These sounds are not similar to anthropogenic or geophysical sounds [55,56], but have characteristics of biological sounds, specifically baleen whale vocalizations. First, the unidentified tonal call was 5 seconds long and had a low frequency component that was often repeated. It was similar to blue whale vocalizations, but blue whale vocalizations’ duration is typically much longer (10–20 s) [31] than the unidentified tonal sound. It was, however, of similar duration as the blue whale CNP tonal reported here. Given its distinct frequency, however, and generally high level of blue whale frequency synchrony within a population [57,58], as well as a lack of consistent sequences of these tonals that would be indicative of a song, we separated it into its own call type. This call peaked during summer months at Tinian.

Secondly, the unidentified pulsed sound was a series of pulses that differed from other reported calls in the study. This pulsed sound contained consecutive pulsing sequences that were spaced about 5 seconds apart. Unfortunately, most of them had low signal-to-noise ratio, thus it was challenging to measure the features of this call very accurately, which may be part of the reason for the large variation in the start frequency of the sound. The unidentified pulsed sounds were distinct, however, due to their clear repetitive structure. Given the relatively low sample size we encountered for these calls and the small geographical scope of this study, we do not have enough information to try to infer possible sources of these two call types. Ideally, recording of these calls in the presence of an animal would be used to confirm the identity of the caller. In absence of visual confirmations, caller identity might be inferred by documenting the signal’s occurrence across different regions of the North Pacific, and possibly beyond. More extensive information on spatial and temporal occurrence of these sounds could then be used to hypothesize on their potential sources [59]. The quantitative and qualitative description of the sounds we provide here should be helpful for these future investigations.

## Limitations of our study

Passive acoustic monitoring provides opportunities for tracking marine mammals over large spatial and temporal scales, but it also has certain limitations. The presence of noise, including instrument self-noise, masked calls in the recordings and caused a reduction of the effort in logging baleen whale calls. At this time, there is no efficient way to remove noise [60] to allow better focusing on clear recordings. Reduction of the recording effort due to masking from noise may have reduced the possibility of detecting the presence of some calls. This would have most affected our ability to determine the seasonality and occurrence of calls that are not frequently produced, such as calls of unknown origin or fin whale 40 Hz calls. On the other hand, it could have resulted in higher peaks of presence during times with higher effort, like the summer peak in CNP tonals. In addition, acoustic data can only provide information on calling animals. Absence of calls does not indicate absence of an animal; an animal could be present, but not calling in which case it would not be detected by our methods.

Another challenge was that most of the calls were not high intensity, meaning the source was likely quite distant from the recorder. When the calls are low intensity, the identification of the call can become difficult. The low intensity of the calls, revealing their presence farther offshore, increases the challenges in trying to identify animals producing these call types when the source is unknown.

## Conclusions

We found five distinct sounds produced by known or presumed baleen whales in recordings off Saipan and Tinian. We document higher occurrences of blue and fin whale acoustic presence at Tinian, with more calls also generally recorded in the winter. Two new, previously unreported sounds were described in the study and were likely of biological origin, one tonal and one pulsed sound. The former was more common at Tinian and the later at Saipan. By providing their acoustic features and their occurrence, we hope to help with future identification of the source of these sounds. As this is a remote, difficult to study region, passive acoustic, long-term monitoring stations are a useful means of surveying the region. By describing the baleen whale vocalization repertoire and identifying region-specific call types, we provided support for future studies of population distribution of these species that can be helpful for their conservation. Future research in this region focusing on determining breeding grounds, better understanding seasonality of calls in the region, and identifying sources of unknown calls described in this study would greatly contribute to current knowledge of this sparsely-studied area.

## Supporting information

S1 Fig Examples of LTSAs used for logging. Examples of one-hour long-term spectral average (LTSA) views that were used for reviewing and logging whale calls: blue whale tonals (top panel), fin whale 40 Hz calls (middle panel), and unidentified tonal sounds (bottom panel).
(JPG)

S1 Checklist Checklist on inclusivity in global research. (PDF)

S2 File Širović [Unpublished]. Estimating propagation distance for blue and fin whale calls at HARP sites at Saipan and Tinian. Description of propagation modeling conducted to estimate approximate detection ranges for blue and fin whale calls at the two recording sites.
(PDF)

S3 File Logs of whale call occurrence in recordings from Saipan and Tinian used in this study. Excel spreadsheets containing logging information for each site: species and type of call logged, as well as date and time of its occurrence. Full data on which logging was conducted can be accessed from NOAA National Centers for Environmental Information: NOAA Pacific Islands Fisheries Science Center: Pacific Islands Passive Acoustic Network (PIPAN) 10kHz Data. https://doi.org/10.25921/Z787-9Y54.
(ZIP)

## References

[1] Mate BR, Lagerquist BA, Calambokidis J. Movements of North Pacific blue whales during the feeding season off southern california and their Southern fall migration1. Marine Mammal Science. 1999;15(4):1246–57. doi: 10.1111/j.1748-7692.1999.tb00888.x

[2] Reilly SB, Thayer VG. Blue whale (Balaenoptera Musculus)distribution in the Eastern Tropical Pacific. Marine Mammal Science. 1990;6(4):265–77. doi: 10.1111/j.1748-7692.1990.tb00357.x

[3] Abrahms B, Hazen EL, Aikens EO, Savoca MS, Goldbogen JA, Bograd SJ, et al. Memory and resource tracking drive blue whale migrations. Proc Natl Acad Sci U S A. 2019;116(12):5582–7. doi: 10.1073/pnas.1819031116 30804188 PMC6431148

[4] Ingman K, Hines E, Mazzini PLF, Rockwood RC, Nur N, Jahncke J. Modeling changes in baleen whale seasonal abundance, timing of migration, and environmental variables to explain the sudden rise in entanglements in California. PLoS One. 2021;16(4):e0248557. doi: 10.1371/journal.pone.0248557 33857163 PMC8049321

[5] Bannister JL. Baleen Whales (Mysticeti). Encyclopedia of Marine Mammals. Elsevier. 2018. p. 62–9. doi: 10.1016/b978-0-12-804327-1.00058-3

[6] Jefferson TA, Webber MA, Pitman RL. Marine Mammals of the World: A Comprehensive Guide to Their Identification. Elsevier. 2008.

[7] Rice DW. Marine mammals of the world: systematics and distribution. Allen Press. 1998.

[8] Fiedler PC, Redfern JV, Forney KA, Palacios DM, Sheredy C, Rasmussen K. Prediction of large whale distributions: A comparison of presence-absence and presence-only modeling techniques. Frontiers in Marine Science. 2018. doi: 10.3389/fmars.2018.004199

[9] Mate BR, Lagerquist BA, Calambokidis J. Movements of North Pacific blue whales during the feeding season off Southern California and their southern fall migration. Mar Mamm Sci. 1999;15(4):1246–57.

[10] Mizroch SA, Rice DW, Zwiefelhofer D, Waite J, Perryman WL. Distribution and movements of fin whales in the North Pacific Ocean. Mammal Review. 2009;39(3):193–227. doi: 10.1111/j.1365-2907.2009.00147.x

[11] Stafford KM. Two types of blue whale calls recorded in the Gulf of Alaska. Marine Mammal Science. 2003;19(4):682–93. doi: 10.1111/j.1748-7692.2003.tb01124.x

[12] Zerbini AN, Waite JM, Laake JL, Wade PR. Abundance, trends and distribution of baleen whales off Western Alaska and the central Aleutian Islands. Deep Sea Research Part I: Oceanographic Research Papers. 2006;53(11):1772–90. doi: 10.1016/j.dsr.2006.08.009

[13] Oleson EM, Wiggins SM, Hildebrand JA. Temporal separation of blue whale call types on a southern California feeding ground. Animal Behaviour. 2007;74(4):881–94. doi: 10.1016/j.anbehav.2007.01.022

[14] Širovic A, Rice A, Chou E, Hildebrand J, Wiggins S, Roch M. Seven years of blue and fin whale call abundance in the Southern California Bight. Endang Species Res. 2015;28(1):61–76. doi: 10.3354/esr00676

[15] Gardner JV, Armstrong AA, Calder BR, Beaudoin J. So, How Deep Is the Mariana Trench?. Marine Geodesy. 2014;37(1):1–13. doi: 10.1080/01490419.2013.837849

[16] Hill MC, Oleson EM, Bradford AL, Martien KK, Steel D, Baker JS. Assessing cetacean populations in the Mariana Archipelago: A summary of data and analyses arising from Pacific Islands Fisheries Science Center Surveys from 2010 to 2019. NOAA Technical Memorandum. 2020. doi: 10.25923/wrye-6h14

[17] Fulling GL, Thorson PH, Rivers J. Distribution and Abundance Estimates for Cetaceans in the Waters off Guam and the Commonwealth of the Northern Mariana Islands. Pacific Science. 2011;65(3):321–43. doi: 10.2984/65.3.321

[18] Hill M, Bradford A, Steel D, Baker C, Ligon A, Ü A, et al. Found: a missing breeding ground for endangered western North Pacific humpback whales in the Mariana Archipelago. Endang Species Res. 2020;41:91–103. doi: 10.3354/esr01010

[19] Clark CW. Acoustic Behavior of Mysticete Whales. Sensory Abilities of Cetaceans. Springer US. 1990. p. 571–83. doi: 10.1007/978-1-4899-0858-2_40

[20] Oleson E, Calambokidis J, Burgess W, McDonald M, LeDuc C, Hildebrand J. Behavioral context of call production by eastern North Pacific blue whales. Mar Ecol Prog Ser. 2007;330:269–84. doi: 10.3354/meps330269

[21] McDonald M, Mesnick SL, Hildebrand JA. Biogeographic characterization of blue whale song worldwide: using song to identify populations. J Cetacean Res Manag. 2006;8:1.

[22] Širović A, Oleson EM. The Bioacoustics of Blue Whales—Global Diversity and Behavioral Variability in a Foraging Specialist. Ethology and Behavioral Ecology of Marine Mammals. Springer International Publishing. 2022. p. 195–221. doi: 10.1007/978-3-030-98449-6_9

[23] McDonald MA, Calambokidis J, Teranishi AM, Hildebrand JA. The acoustic calls of blue whales off California with gender data. J Acoust Soc Am. 2001;109(4):1728–35. doi: 10.1121/1.1353593 11325141

[24] Paniagua‐Mendoza A, Gendron D, Romero‐Vivas E, Hildebrand JA. Seasonal acoustic behavior of blue whales (Balaenoptera musculus) in the Gulf of California, Mexico. Marine Mammal Science. 2016;33(1):206–18. doi: 10.1111/mms.12362

[25] Mellinger DK, Clark CW. Blue whale (Balaenoptera musculus) sounds from the North Atlantic. J Acoust Soc Am. 2003;114(2):1108–19. doi: 10.1121/1.1593066 12942988

[26] Rankin S, Ljungblad D, Clark C, Kato H. Vocalisations of Antarctic blue whales, Balaenoptera musculus intermedia, recorded during the 2001/2002 and 2002/2003 IWC/SOWER circumpolar cruises, Area V, Antarctica. J Cetacean Res Manag. 2005;7:1.

[27] Schall E, Di Iorio L, Berchok C, Filún D, Bedriñana‐Romano L, Buchan SJ, et al. Visual and passive acoustic observations of blue whale trios from two distinct populations. Marine Mammal Science. 2019;36(1):365–74. doi: 10.1111/mms.12643

[28] Oestreich WK, Fahlbusch JA, Cade DE, Calambokidis J, Margolina T, Joseph J, et al. Animal-Borne Metrics Enable Acoustic Detection of Blue Whale Migration. Curr Biol. 2020;30(23):4773-4779.e3. doi: 10.1016/j.cub.2020.08.105 33007246

[29] Rice A, Debich AJ, Širović A, Oleson EM, Trickey JS, Varga LM, et al. Cetacean occurrence offshore of Washington from long-term passive acoustic monitoring. Mar Biol. 2021;168(8). doi: 10.1007/s00227-021-03941-9

[30] Rice A, Širović A, Trickey JS, Debich AJ, Gottlieb RS, Wiggins SM, et al. Cetacean occurrence in the Gulf of Alaska from long-term passive acoustic monitoring. Mar Biol. 2021;168(5). doi: 10.1007/s00227-021-03884-1

[31] Stafford KM, Nieukirk SL, Fox CG. Geographic and seasonal variation of blue whale calls in the North Pacific. J Cetacean Res Manag. 2001. doi: 10.475336/jcrm.v3il.902

[32] Thompson PO, Findley LT, Vidal O, Cummings WC. Underwater sounds of blue whales, Balaenoptera Musculus, in the Gulf of California, Mexico. Marine Mammal Science. 1996;12(2):288–93. doi: 10.1111/j.1748-7692.1996.tb00578.x

[33] Širović A, Oleson EM, Favilla A, Fisher-Pool P. Blue whale song variability in the North Pacific Ocean. In: 2016.

[34] Stafford KM, Nieukirk SL, Fox CG. Low-frequency whale sounds recorded on hydrophones moored in the eastern tropical Pacific. J Acoust Soc Am. 1999;106(6):3687–98. doi: 10.1121/1.428220 10615707

[35] McDonald M, Širović A, Sugioka H, Kato H, Yoshida R, Kyo N. Preliminary analysis of blue and fin whale acoustic presence off Hokkaido Japan. Paper SC/67A/NH01 presented at the IWC Scientific Committee. 2017. Available from: secretariat@iwc.int

[36] Watkins WA. Activities and underwater sounds of fin whales. Sci Rep Whales Res Inst. 1981;33:83–117.

[37] Sirović A, Williams LN, Kerosky SM, Wiggins SM, Hildebrand JA. Temporal separation of two fin whale call types across the eastern North Pacific. Mar Biol. 2013;160(1):47–57. doi: 10.1007/s00227-012-2061-z 24391281 PMC3873066

[38] Castellote M, Clark CW, Lammers MO. Fin whale (Balaenoptera physalus) population identity in the western Mediterranean Sea. Marine Mammal Science. 2011;28(2):325–44. doi: 10.1111/j.1748-7692.2011.00491.x

[39] Delarue J, Todd SK, Van Parijs SM, Di Iorio L. Geographic variation in Northwest Atlantic fin whale (Balaenoptera physalus) song: implications for stock structure assessment. J Acoust Soc Am. 2009;125(3):1774–82. doi: 10.1121/1.3068454 19275334

[40] Oleson EM, Širović A, Bayless AR, Hildebrand JA. Synchronous seasonal change in fin whale song in the North Pacific. PLoS One. 2014;9(12):e115678. doi: 10.1371/journal.pone.0115678 25521493 PMC4270802

[41] Širović A, Oleson EM, Buccowich J, Rice A, Bayless AR. Fin whale song variability in southern California and the Gulf of California. Sci Rep. 2017;7(1):10126. doi: 10.1038/s41598-017-09979-4 28860617 PMC5579205

[42] Stafford KM, Mellinger DK, Moore SE, Fox CG. Seasonal variability and detection range modeling of baleen whale calls in the Gulf of Alaska, 1999-2002. J Acoust Soc Am. 2007;122(6):3378–90. doi: 10.1121/1.2799905 18247747

[43] Watkins W, Daher MA, Reppucci G, George J, Martin D, DiMarzio N, et al. Seasonality and Distribution of Whale Calls in the North Pacific. oceanog. 2000;13(1):62–7. doi: 10.5670/oceanog.2000.54

[44] Croll DA, Clark CW, Acevedo A, Tershy B, Flores S, Gedamke J, et al. Only male fin whales sing loud songs. Nature. 2002;417(6891):809. doi: 10.1038/417809a 12075339

[45] Watkins WA, Tyack P, Moore KE, Bird JE. The 20-Hz signals of finback whales (*Balaenoptera physalus*). The Journal of the Acoustical Society of America. 1987;82(6):1901–12. doi: 10.1121/1.395685 3429729

[46] McDonald MA, Hildebrand JA, Webb SC. Blue and fin whales observed on a seafloor array in the northeast pacific. J Acoust Soc Am. 1995;98(2 Pt 1):712–21. doi: 10.1121/1.413565 7642810

[47] Wiggins SM, Hildebrand JA. High-frequency Acoustic Recording Package (HARP) for broad-band, long-term marine mammal monitoring. In: 2007 Symposium on Underwater Technology and Workshop on Scientific Use of Submarine Cables and Related Technologies, 2007. 551–7. Available from: doi: 10.1109/ut.2007.370760

[48] R Core Team. R: A language and environment for statistical computing. Vienna, Austria: R Foundation for Statistical Computing. 2020.

[49] NOAA National Centers for Environmental Information. 2022: ETOPO 2022 15 Arc-Second Global Relief Model. NOAA National Centers for Environmental Information. [cited 30/03/2024]. Available from: doi: 10.25921/fd45-gt74

[50] Thompson PO, Friedl WA. A long-term study of low frequency sounds from several species of whales off Oahu Hawaii. Cetology. 1982;45.

[51] Stafford KM, Nieukirk SL, Fox CG. An acoustic link between blue whales in the Eastern Tropical Pacific and the Northeast Pacific1. Marine Mammal Science. 1999;15(4):1258–68. doi: 10.1111/j.1748-7692.1999.tb00889.x

[52] Thompson PO, Findley LT, Vidal O. 20-Hz pulses and other vocalizations of fin whales, Balaenoptera physalus, in the Gulf of California, Mexico. The Journal of the Acoustical Society of America. 1990;88(S1):S5–S5. doi: 10.1121/1.2029083 1474220

[53] Moore SE, Stafford KM, Dahlheim ME, Fox CG, Braham HW, Polovina JJ, et al. Seasonal variation in reception of fin whale calls at five geographic areas in the North Pacific. Marine Mammal Science. 1998;14(3):617–27. doi: 10.1111/j.1748-7692.1998.tb00749.x

[54] Archer FI, Rankin S, Stafford KM, Castellote M, Delarue J. Quantifying spatial and temporal variation of North Pacific fin whale (Balaenoptera physalus) acoustic behavior. Marine Mammal Science. 2019;36(1):224–45. doi: 10.1111/mms.12640

[55] Hildebrand J. Anthropogenic and natural sources of ambient noise in the ocean. Mar Ecol Prog Ser. 2009;395:5–20. doi: 10.3354/meps08353

[56] Pijanowski BC, Villanueva-Rivera LJ, Dumyahn SL, Farina A, Krause BL, Napoletano BM, et al. Soundscape Ecology: The Science of Sound in the Landscape. BioScience. 2011;61(3):203–16. doi: 10.1525/bio.2011.61.3.6

[57] McDonald M, Hildebrand J, Mesnick S. Worldwide decline in tonal frequencies of blue whale songs. Endang Species Res. 2009;9:13–21. doi: 10.3354/esr00217

[58] Carbaugh-Rutland A, Have Rasmussen J, Sterba-Boatwright B, Širović A. Geographically distinct blue whale song variants in the Northeast Pacific. Endang Species Res. 2021;46:19–33. doi: 10.3354/esr01145

[59] Baumann-Pickering S, Roch MA, Brownell RL Jr, Simonis AE, McDonald MA, Solsona-Berga A, et al. Spatio-temporal patterns of beaked whale echolocation signals in the North Pacific. PLoS One. 2014;9(1):e86072. doi: 10.1371/journal.pone.0086072 24465877 PMC3899217

[60] Wimmer J, Towsey M, Roe P, Williamson I. Sampling environmental acoustic recordings to determine bird species richness. Ecol Appl. 2013;23(6):1419–28. doi: 10.1890/12-2088.1 24147413

