# Supplementary figures and images for: Blue and fin whales in the Northern Mariana Islands: Their call characteristics and occurrence
Source: PLoS One. 2025 Aug 21;20(8):e0329398. doi: 10.1371/journal.pone.0329398 (PMC12370069; doi:10.1371/journal.pone.0329398)

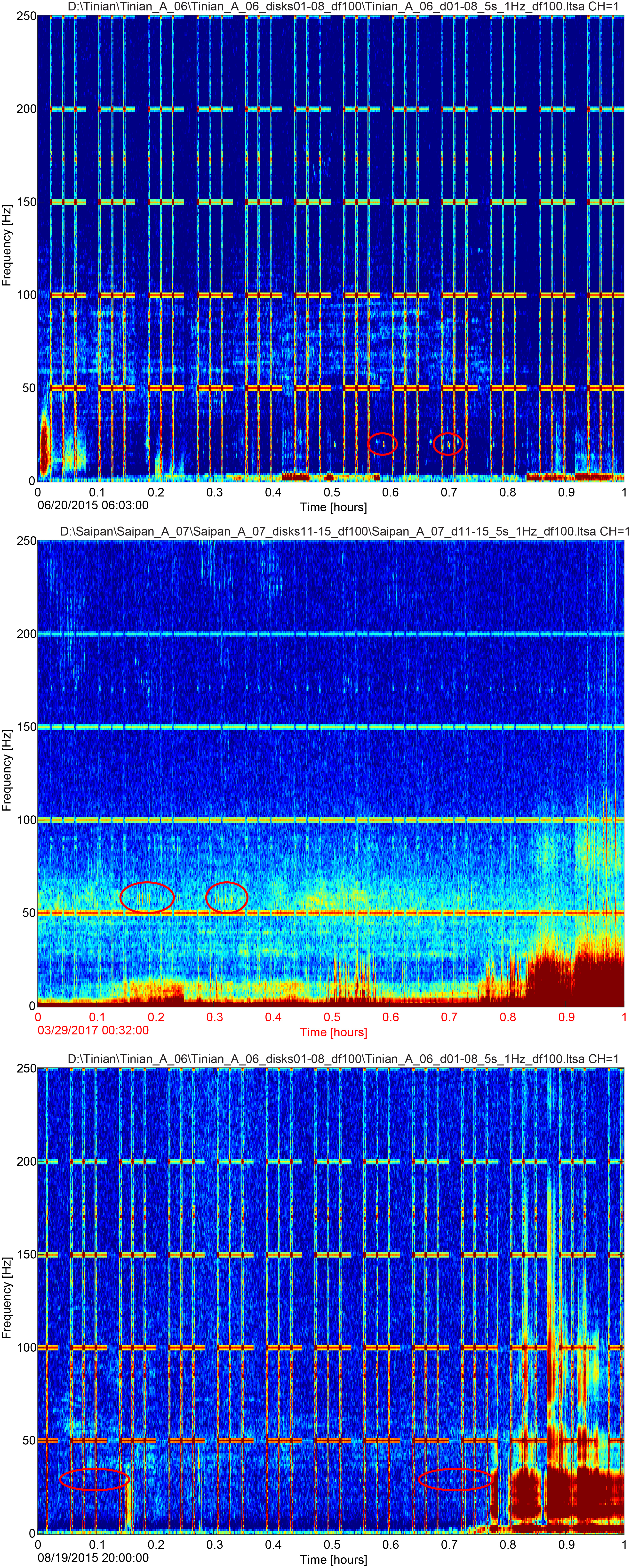

Supplement: S1 Fig — Examples of one-hour long-term spectral average (LTSA) views that were used for reviewing and logging whale calls: blue whale tonals (top panel), fin whale 40 Hz calls (middle panel), and unidentified tonal sounds (bottom panel). (JPG) [file pone.0329398.s001.jpg]
