# Supplementary material for: Blue and fin whales in the Northern Mariana Islands: Their call characteristics and occurrence
Source: PLoS One. 2025 Aug 21;20(8):e0329398. doi: 10.1371/journal.pone.0329398 (PMC12370069; doi:10.1371/journal.pone.0329398)
Supplement: S2 File — Description of propagation modeling conducted to estimate approximate detection ranges for blue and fin whale calls at the two recording sites. (PDF) [file pone.0329398.s003.pdf]

Estimating propagation distance for blue and fin whale calls at HARP sites at Saipan and Tinian
By A. Širović

Approach

To estimate distances to calling whales, information on (1) call source levels (SL), (2) received levels (RL), and (3) transmission loss (TL) in the studied environment are required. As TL models were run for single frequencies characteristics of different calls, all SL and RL assumptions and measures were conducted for the appropriate frequencies. Since our knowledge of call SLs is relatively poor in general, and almost non-existent for calls produced in this particular region, the estimates I used for this analysis were based on the best-available published records. As there are no SL estimates for tonal Western Pacific blue whale call type, I used 186 dB re: 1µPa @ 1 that was estimated for relatively similar tonal calls of Eastern Pacific blue whales (McDonald et al. 2001). For fin whale 20 Hz calls I adjusted Weirathmueller et al. (2013) levels for the average 5 Hz bandwidth of fin whale calls (Watkins 1981, Watkins et al. 1987) and used 182 dB re: 1 µPa @ 1 m at 20 Hz.

Received levels were measured based on 1 s of data at the appropriate frequency for each species and call type. Thus for blue and fin whales, I measured RL at 20. A subset of three calls from each calling sequence of blue whale tonals and fin whale 20 Hz calls was used to estimate the average RL for that calling bout. In an effort to minimize overrepresentation of calls from a single animal, I used sequences that were separated by at least 2 or more hours. The logged start times from this subset of calls were used to extract 1 s of data and estimate the RL at the appropriate frequency.

Finally, I developed TL models for low frequency sounds at the two HARP deployment locations using EMSE Workbench (Boston University) framework. To develop these models, I used environmental data (0.5 degree resolution bathymetry, 0.1 degree resolution sediment properties data, and monthly sound speed and wind profiles) available in ESME. I developed separate models for each species' call types of interest over four seasons. I used January data as representative of winter, April for spring, July for summer, and October for fall. By using parabolic equation modeling, I calculated TL along 16 radials centered at the HARP deployment location out to the distances of 100 km in 495 m bin increments. In all cases, I assumed whales were calling at 30 m depth.

Based on assumed whale call SLs described above, and measured RL, I used these models to estimate ranges over which baleen whale calls can be detected at the two sites. This was done by calculating the TL of each species using the formula: TL = SL – RL. Then a set of ranges at which that calling animal could be was pulled out of the models based on ranges at which the calculated TL occurred. The distributions of modeled ranges given our measured RLs are given for each species and site where they were recorded.

Results

A total of one blue whale tonal and 10 fin whale calling sequences were sampled and RL was measured at the Saipan site (Figure 1). From the Tinian site, six blue whale and two fin whale sequences were measured (Figure 2).

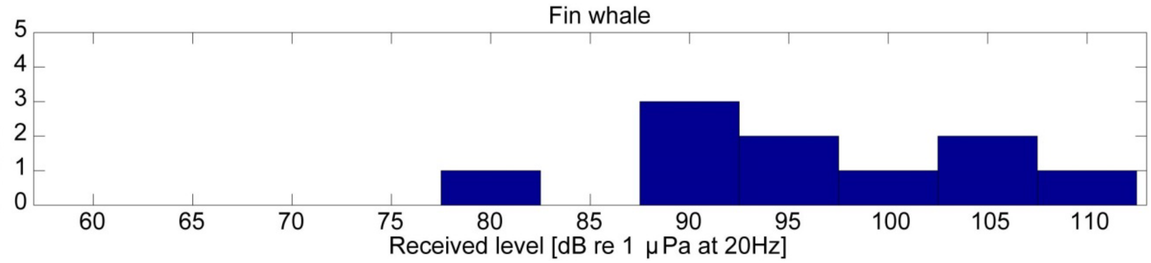

Figure 1. Frequency distribution of measured received levels at appropriate frequency for a sample of fin whale calls recorded at Saipan.

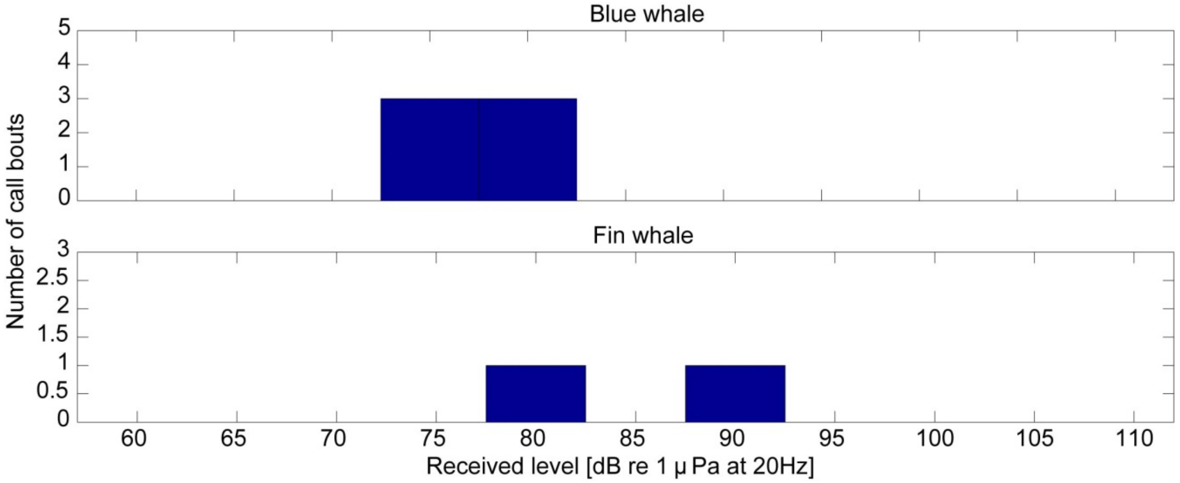

Figure 2. Frequency distribution of measured received levels at 20 Hz for a sample of blue (top) and fin whale calls (bottom) recorded at Tinian.

At Saipan, calling fin whales may have occurred over a range of distances, from as close to 10 or 20 km to potentially even beyond our modeled detection range (100 km). The one blue whale call sequence I measured was likely from a relatively distant whale (farther than 60 km).

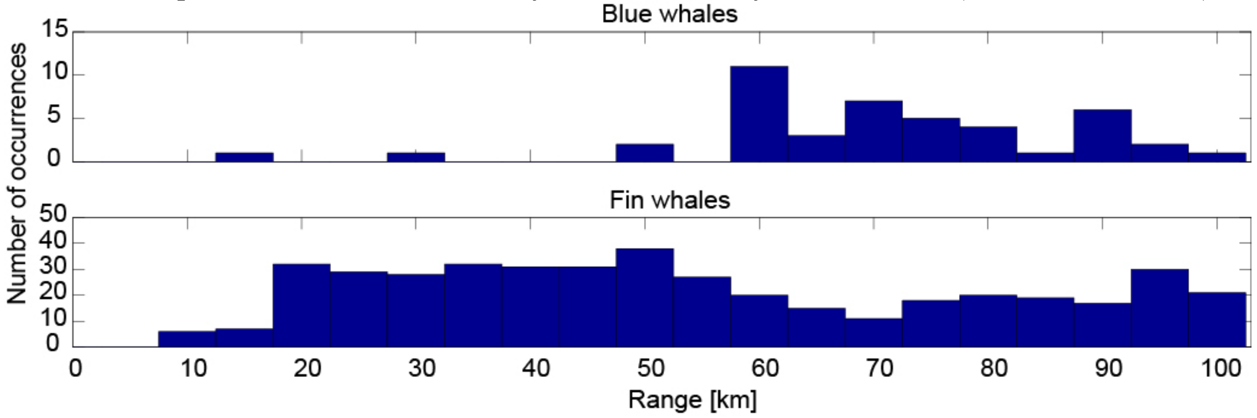

Figure 3. Distribution of possible range estimates based on measured RL and modeled TL for calling blue (top) and fin whales (bottom) recorded at Saipan.

At Tinian, there was a lot less variation in range distributions in the measured examples of blue and fin whales, with both species likely calling at a distance of more than 20 km, but mostly closer than 100 km (Figure 4).

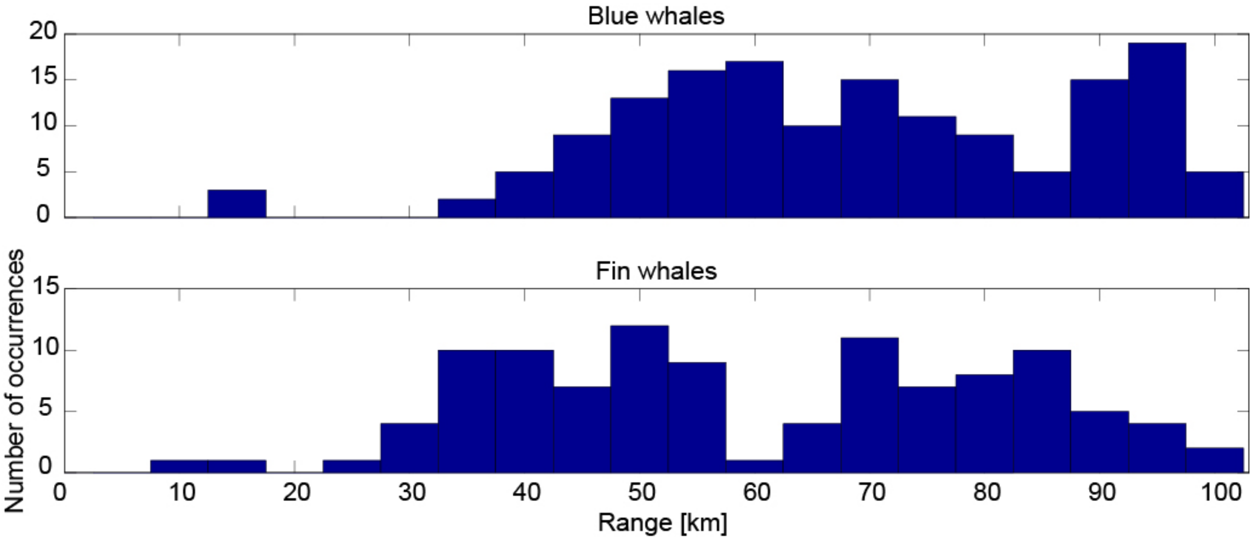

Figure 4. Distribution of possible range estimates based on measured RL and modeled TL for calling blue (top) and fin whale (bottom) recorded at Tinian.

All results presented here only show propagation models for April since spring was the period when most calls occurred. Differences at other times on average were between 1 and 3 dB, with generally less TL (indicating a somewhat larger detection range) occurring in January than April, and with most TL and shorter detection ranges in July.

References

Gedamke J, Costa DP, Dunstan A (2001) Localization and visual verification of a complex minke whale vocalization. Journal of the Acoustical Society of America 109:3038-3047

McDonald MA, Calambokidis J, Teranishi AM, Hildebrand JA (2001) The acoustic calls of blue whales off California with gender data. Journal of the Acoustical Society of America 109:1728-1735

Richardson WJ, Greene Jr., CR, Malme CI, Thomson DH (1995) Marine mammals and noise. Academic Press, San Diego, CA.

Watkins WA (1981) Activities and underwater sounds of fin whales. Scientific reports of the Whales Research Institute 33:83-117

Watkins WA, Tyack P, Moore KE, Bird JE (1987) The 20-Hz signal of finback whales (*Balaenoptera physalus*). The Journal of the Acoustical Society of America 82:1901 - 1912

Weirathmueller MJ, Wilcock WSD, Soule DC (2013) Source levels of fin whale 20 Hz pulses measured in the Northeast Pacific Ocean. Journal of the Acoustical Society of America 133:741-749
